# Variation of Low-Frequency Time-Code Signal Field Strength during the Annular Solar Eclipse on 21 June 2020: Observation and Analysis

**DOI:** 10.3390/s21041216

**Published:** 2021-02-09

**Authors:** Xin Wang, Bo Li, Fan Zhao, Xinyu Luo, Luxi Huang, Ping Feng, Xiaohui Li

**Affiliations:** 1 National Time Service Center, Chinese Academy of Sciences, Xi’an 710600, China; libo@ntsc.ac.cn (B.L.); zhaofan@ntsc.ac.cn (F.Z.); luoxinyu@ntsc.ac.cn (X.L.); huangluxi@ntsc.ac.cn (L.H.); pingfp@ntsc.ac.cn (P.F.); xiaohui@ntsc.ac.cn (X.L.); 2 School of Astronomy and Space Science, University of Chinese Academy of Sciences, Beijing 100049, China; 3 Fengkai Low-Frequency Time-Code Time Service Station, Zhaoqing 526500, China

**Keywords:** annual solar eclipse, low-frequency time-code signal, ionosphere, signal field strength measurement

## Abstract

Due to the occlusion of the moon, an annular solar eclipse will have an effect on the ionosphere above the earth. The change of the ionosphere, for the low-frequency time-code signal that relies on it as a reflection medium for long-distance propagation, the signal field strength, and other parameters will also produce corresponding changes, which will affect the normal operation of the low-frequency time-code time service system. This paper selects the solar eclipse that occurred in China on 21 June 2020, and uses the existing measurement equipment to carry out experimental research on the low-frequency time-code signal. We measured and analyzed the signal field strength from 20 June 2020 to 23 June 2020, and combined solar activity data, ionospheric data, and geomagnetic data, and attempted to explore the reasons and rules of the change of signal parameters. The results showed that the field strength of the low-frequency time-code signal changed dramatically within a short time period, the max growth value can reach up to 17 dBμV/m and the variation trend yielded ‘three mutations’. This change in signal field strength is probably due to the occurrence of a solar eclipse that has an effect on the ionosphere. When the signal propagation conditions change, the signal strength will also change accordingly.

## 1. Introduction

Owing to the close relationship between the Earth and the Sun, the solar eclipse observation has been very important not only for the study of astrophysics and the sun itself [1], but also for the study of radio transmissions that rely on the ionosphere as a propagation and reflection medium [2,3]. When a solar eclipse occurs, owing to the moon’s shielding, the intensity of solar light weakens, resulting in significant changes in the field strength, phase, and other parameters of radio signals (especially low-frequency signals) that rely on the low ionosphere for long-distance transmission [4,5]. Therefore, based on the important influence of the solar eclipse on low-frequency signal propagation, researchers have observed and analyzed the variation of low-frequency signal parameters at the receiving point, explored the relationship between this variation and the ionosphere, and deduced the influencing mechanism between the solar eclipse and the ionosphere.

Several scholars have studied the impact of the eclipse based on the evaluation of very low-frequency (VLF) signals or low-frequency (LF) signals. As reported by Vishal Chauhan and Birbal Singh, 2010 [6], VLF signal amplitude values (f = 19.8 kHz) were analyzed 7 days before and 7 days after the total solar eclipse of 22 July 2009, to allow pre- and post-event comparisons. According to this report, on 22 July the signal amplitude value was the lowest. This was attributed to the fact that when the total solar eclipse occurred, interactions between D electrons and neutral particles in the ionosphere caused a decrease in the concentration of the ionosphere. Wenzel et al., 2017 [7] observed the solar eclipse that occurred on 20 March 2015, focused on the amplitude value of VLF signals at short distances at different frequency points within the range of 25–80 kHz, and modeled them with the long-wavelength propagation capability (LWPC) model. De et al., 2011 [8] observed the amplitude and phase of the VLF signal for 10 days during the solar eclipse (5 days before and 5 days after the event) which occurred on 22 July 2009, and compared the results with the theoretical values obtained with the LWPC model. The amplitude of the signal decreased after the event compared with its values 5 days before its occurrence, and a minimum decrease in its value of 2.5 dB was documented. Clilverd et al., 2001 [9] observed the amplitude of the VLF signal based on five test points set to explore the influence of the total solar eclipse on the low-frequency signal on 11 August 1999. HOY et al., 1970 [10] observed phase of the VLF signal during two solar eclipses was reported, and concluded that the solar eclipse would produce a phase delay similar to that produced at night with a standard deviation of 2 μs. V. I. Kozlov et al., 2007 [11] studied the influence of the VLF signal during the solar eclipse on 29 March 2006, and showed that the amplitude of the received signal increased by approximately 20%.

The frequent eruptions of various forms of solar activity affect the ionosphere above the earth anytime and anywhere, and may induce complex chemical and dynamic processes to the ionosphere, thus causing changes in its morphology, structure, and dynamic behavior. Among scholars who conducted extensive research and experimentation on the change of the sun’s activity on the ionosphere, Norsuzila Ya’acob et al., 2011 [12] focused on the responses of the ionospheres of Indonesia and Singapore of the solar eclipse on 26 January 2009. Total electron content (TEC) data were obtained over a period of three days (1 day before, during, and 1 day after the eclipse), and were analyzed. The results showed that during the solar eclipse, the TEC level decreased owing to the shrinkage of the ionizing radiation. Aplin et al., 2016 [13] summarized the atmospheric changes related to 44 solar eclipses and studied the changes in the meteorological parameters during solar eclipses. Jung-Hee Kim et al., 2018 [14] measured the gravity and magnetic field during the solar eclipse and compared it with the results from the same location during the solar eclipse on 11 August 1999. Zhang et al., 2010 [15] used the study on the dynamic process of the ionosphere over China during the partial eclipse of 11 August 2018, as an example, and found that the electron density of the ionosphere exhibited a quasi-periodic oscillation with amplitudes in the range of 9–14% with a maximum decrease of 26%.

As the only time service method strongly recommended by the International Telecommunication Union (ITU), because of the low-power consumption and convenience of use of the terminal equipment, the low-frequency time-code time service technology has huge development prospects. However, during the period of the annular eclipse, the transmission mechanism and the variation of signal parameters of low-frequency time-code signal still remain unknown, and the lack of relevant experimental and measurement data has stalled these research endeavours. Therefore, this article takes the low-frequency time-code signal as an example to conduct experimental research on the low-frequency time-code signal which was transmitted from Shangqiu Station (Shangqiu, Henan Province, China), and uses the existing signal field strength measurement system to measure the signal field strength data generated during the solar eclipse on 21 June 2020 at Sanya (Sanya, Hainan Province, China). Accordingly, to explore the changing rules of signal parameters, signal field strength data were used in combination with the ionospheric, solar activity, and geomagnetic data, during the annular eclipse. This study evaluates the influence of the annular eclipse on the field strength of low-frequency time-code signals; analyzes the influence mechanism to improve the stability and continuity of the low-frequency time-code time service system; and meets the application requirements of time-frequency signals in space science, aerospace, and other related technology industries.

## 2. Background and Observations

### 2.1. Annular Eclipse

The process of annular eclipse is divided into the initial, partial eclipse, beginning of annular eclipse, eclipse, end of annular eclipse, partial eclipse, and final contact. In essence, the annular eclipse happens when the Moon and Sun are precisely in line, but the Moon is relatively far from Earth (closer to apogee) and therefore appears smaller than the Sun and cannot create a total eclipse. Therefore, the Sun appears as a very vivid ring, or annulus, during the eclipse maxima [12,16]. During an annular eclipse, the magnitude of eclipse increases and the solar zenith distance decreases from the initial depletion to the eclipse. These two factors affect the change of solar radiation flux to the ionosphere [17]. Specifically, the electron density (assumed to be an exponential mode) gradient (greater than the response value of the reference day) of the D-region increases [18], the electron density increases, the reflection coefficient increases, radio signals are enhanced, and the field strength value is greater than the reference day value. Additionally, before and after the eclipse, the magnitude of the eclipse increases to a maximum value and then slowly decreases, and the solar zenith distance, electron density gradient, electron density, and the reflection coefficient all decrease [19]. The signal drops from the maximum to the minimum value, then slowly changes, and gradually returns to the circular eclipse state at the corresponding time of the reference day.

### 2.2. Low-Frequency Time-Code Signal Propagation Principle

**Figure 1:**
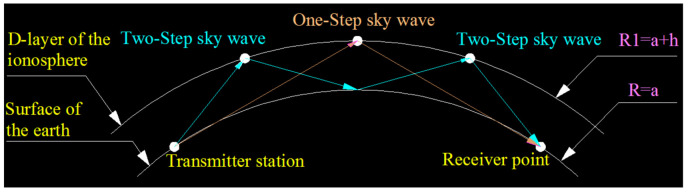
Sky wave propagation path of the low-frequency time-code signal.

The low-frequency time-code signal can be considered as the superposition of sky and ground waves [20]. Subject to the excitation of the transmission antenna on the surface of the earth, radio waves propagate along the surface based on diffraction, and are often referred to as ground waves. The diffraction mechanism shows that the boundary of the underground medium is stimulated by radio waves to induce a current and bound charge, thus generating a secondary radiation field that guides the radio waves to propagate along the surface [21]. Because ground waves propagate along the earth’s surface, they are affected by many factors in the propagation path, such as the relative dielectric constant, earth conductivity, distance of transceiver point and topography, and others, but they have no association with the ionosphere and solar activity. In contrast to the ground wave propagation mechanism, the sky wave of the low-frequency time-code signal is reflected by the ionosphere to propagate. Owing to the longer wavelength and the good conductive properties of the ionospheric D-region, when the sky wave is reflected and propagated from the D-region at the lower boundary of the ionosphere, the signal is transmitted for a long distance with one or more jumps. Figure 1 shows the sky wave signal propagation path.

#### 2.2.1. Ground Wave Area

For low-frequency time-code signal, the propagation field strength of the ground wave signals can be expressed as

(1) $$E(\mathrm{dB})=109.54+A-20\mathrm{lg}d+10\mathrm{lg}P_{\sum }$$

where *d* is the spatial path of the wave propagation, *A* is the attenuation factor of the ground wave (dB), and $P_{\sum }$ is the radiation power of the transmitting antenna in kW. The basic field is $E^{’}=109.54+A-20\mathrm{lg}d$, that is, the field strength at the receiving point when $P_{\sum }$ is 1 kW.

#### 2.2.2. Sky Wave Area

For low-frequency time-code signals, the propagation field strength of the sky wave signals can be expressed as

(2) $$E=\frac{0.6p_{t}{}^{\frac{1}{2}}R_{i}{}^{m}R_{g}{}^{m-1}D_{m}D_{g}{}^{m-1}F_{t}F_{r}{\left(\cos^{2}\psi \right)}^{m}}{L}$$

where *p_t_* is the antenna radiation power, *R_i_* is the ionosphere reflection coefficient, *R_g_* is the ground emission coefficient, *D_m_* is the ionospheric focus factor, *D_g_* is the spherical divergence factor, *F_t_* is the transmitting antenna factor, *F_r_* is the receiving antenna factor, *ψ* is the launch angle and arrival angle of the sky wave on the ground relative to the horizon, and *L* is length of the sky wave propagation ray.

### 2.3. Observations

**Figure 2:**
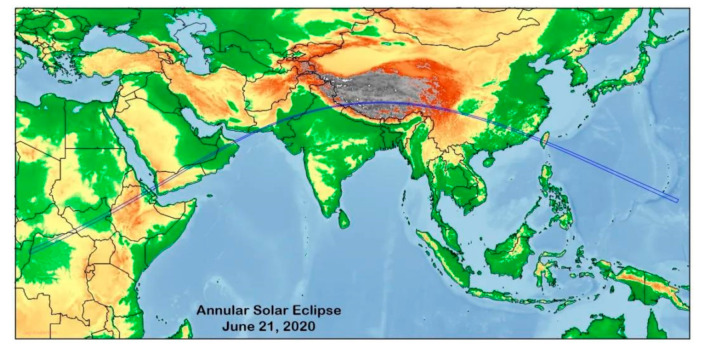
Annular eclipse path on 21 June 2020. (source: http://tech.sina.com.cn/d/s/2020-06-17/doc-iircuyvi8955614.shtml?cre=tianyi&mod=pchp&loc=34&r=0&rfunc=46&tj=none&tr=12 (accessed on 6 February 2021)).

The annular solar eclipse on 21 June 2020 is the only one visible in China during the past ten years, and the eclipse was very close to the total eclipse, so the ‘golden ring’ was very thin. The solar eclipse started at 03:46 (UTC) and ended at 09:34 (UTC) in China. The ring eclipse entered China from Tibet’s Holy Lake Mapanyongcuo, passed through the Southern Sichuan Basin, entered Fujian through the northeastern Guizhou and Southern Jiangxi Provinces, and entered the sea at Xiamen, and finally left China across the Taiwan island. Figure 2 shows the path of the annular solar eclipse on 21 June 2020.

To observe the influence of the annular solar eclipse on the low-frequency time-code signal which transmitted from Shangqiu, Henan Province during 20–23 June 2020, a dedicated signal measurement device was used to measure the low-frequency time-code signal in Sanya, Hainan Province, and conducted analyses of the field strength changes of the low-frequency time-code signal, and explored the change process of the low-frequency time-code signal during the occurrence of the solar eclipse.

## 3. Materials and Methods

### 3.1. Test Point Selection Strategy

Owing to the fact that the low-frequency time-code signal relies on sky waves for long-distance transmission, if we want to explore the influence of the solar eclipse on the low-frequency time-code signal, the signal reflection point needs to be set in the ring eclipse zone, the signal from the Shangqiu station of the low-frequency time code is reflected on the test point by a certain point on the eclipse zone. At this time, the ionospheric changes over the eclipse zone will affect the sky wave of the low-frequency time code signal. Therefore, the setting of the test point needs to be at the mirror point of Shangqiu Station outside the ring eclipse zone. Based on repeated searches and verification, this study selected Sanya of the Hainan Province as the test site, whose sky wave reflection point was located in Hengyang of the Hunan Province. Table 1 shows several important time points of the solar eclipse.

### 3.2. Data Source

**Figure 3:**
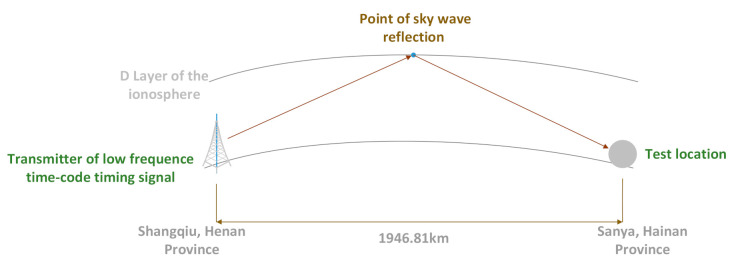
Design of experimental scheme for annular eclipse observations.

To explore the influence of the solar eclipse on the low-frequency time code signal, this paper designed an experimental plan as shown in the Figure 3. When the solar eclipse began, the low-frequency time-code signal which was sent by the transmit station in Shangqiu, Henan Province was reflected by the D-region of the ionosphere and was captured by the signal measurement system in Sanya, Hainan, so that the signal field strength value could be measured.

The signal transmitter and receiver are 1946.81 km apart, the transmission power was 50 kW, the electromagnetic environment around the receiving point was stable, and there were no tall buildings and other shielding objects around the test site.

### 3.3. Field Strength Measurement Principle

As an important parameter for the low-frequency time-code signal, the field strength can directly and truly reflect its spatial strength, and it is also the most important parameter for evaluating the coverage and communication efficiency of a low-frequency time-code signal system.

The field strength of the low-frequency time-code signal is the electromagnetic field strength within a certain space emitted by a high-power antenna [22], and its electric field strength component is commonly used to describe it. In practical engineering applications, the decibel (dB) expression in μV/m is commonly used as the standard, and it is counted as dBμV/m.

(3) $$\mathrm{dB}(\mu V/m)=20\mathrm{lg}\frac{E(\mu V/m)}{1(\mu V/m)},$$

During reception in the presence of an electric field *E* (in the electromagnetic field) [23], the induced voltage on the antenna is *U*, and the relationship between the signal field is expressed by (4) [24]. The effective height *H* of the receiving antenna can be calculated based on antenna theory.

(4) U=H⋅E,

In actual engineering surveys, the receiving antenna should be matched with the measured signal polarization, and the measuring instrument should be impedance matched to ensure maximization of the measurement of the induced signal at the receiving antenna. The signal level value is obtained by the cable that is connected to the measurement instrument [25]. At the same time, considering the receiving antenna and cable loss associated with the signal transfer, the field strength can be expressed as [26]

(5) $$E_{r}=E+K+L,$$

where *E_r_* is the signal field strength, *K* is a factor associated with the receiving antenna, and *L* is the attenuation value of the transmission cable and other accessories.

### 3.4. Experiment Design

When the antenna is in line with the polarisation direction of the measured signal in the air, the antenna will obtain the maximum induced signal. Therefore, the measurement of the low-frequency field strength can be converted into the measurement of the induced signal amplitude. By amplifying and filtering the signal on the antenna, testing and calculating the digitised signal amplitude, selecting an appropriate threshold value, and eliminating outliers and interference data, the field strength of the low-frequency time-code signal can be obtained.

**Figure 4:**
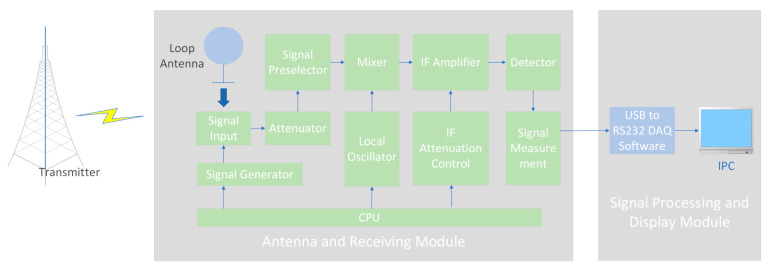
Experimental equipment for annular eclipse observation.

Based on the aforementioned test principles, we built a low-frequency time-code signal measurement system, which was composed of an antenna and a receiving module, and signal processing and display modules, as shown in Figure 4.

The low-frequency time-code signal is amplitude modulated and pulse width modulated simultaneously, the carrier amplitude falls to 10% of the original amplitude at the beginning of per second (except the 59th s). However, in the engineering, the carrier signal strength is chosen as the test object when the amplitude is not falling in per second. The loop magnetic antenna receives the low-frequency time-code signal with a carrier frequency of 68.5 kHz. The signal enters the frequency preselection module after attenuation, multiplies it with the carrier generated by the local crystal oscillator, and then amplifies and filters it into the detector. The detector consists of a multiplier and a low-pass filter. The envelope signal obtained after the detection is sampled and quantized through the A/D module and converted into a digital signal, this digital signal contains the amplitude information of induction signal received by the antenna. Through the calculating by processed module, the signal strength of the measured signal can be obtained.

The signal measurement system used in this experiment consists of a receiving antenna, linear amplifier, non-linear detector, and a display with a time constant.

If the transmission coefficient *G*(*f*) of a linear amplifier is known, then the spectrum function $E_{i}(f)$ of the amplified signal is also known, and the output voltage of the amplifier can be obtained by using the inverse Fourier transform of the spectrum function method. The relevant expression is

(6) $$V_{0}t=2\int_{0}^{\infty }E_{i}(f)\cdot G(f)\cdot \cos[\omega t+\varphi (f)+\varphi_{i}(f)]df,$$

where $\varphi_{i}(f)$ is the phase angle of the spectral function of the input signal, φ(f) is the phase angle of the transmission coefficient of the amplifier.

A proper signal measurement system should be a frequency selector whose passband is narrow enough such that the spectral density of the interference signal is virtually constant within this band. Therefore, Equation (6) can be simplified to

(7) $$V_{0}t=2E_{i}(f_{0})\int_{f1}^{f2}G(f)\cos[\omega t+\varphi (f)]df,$$

Alternatively, it can be written as, $V_{0}t=2E_{i}(f_{0})\cdot g(t)$. In the equation $g(t)=\int_{f1}^{f2}G(f)\cos[\omega t+\varphi (f)]df$, $E_{i}(f_{0})$ is the spectral function of the input signal in the passband, g(t) is the time characteristic of the amplifier, and $f_{1}$ and $f_{2}$ are the upper and lower frequencies of the pass band, respectively.

It can be observed from the Equations that the waveform of the voltage at the output of the amplifier only depends on the performance of the amplifier and the response of the amplitude is proportional to the spectral function of the input signal in the passband. Thus, it can be expressed as the modulated wave with an envelope of A(t),

(8) $$V_{0}t=A(t)\cdot \cos(\omega_{0}t+\varphi_{0}),$$

The detector of the jammer must respond to the A(t) envelope of the output signal. Therefore, an ideal signal measurement system can be perceived as an instrument that measures the parameters related to the output envelope of the narrowband amplifier.

For the peak alignment detector (QP detection mode listed in the instrument), the discharge time constant is much larger than the charge time constant so that it does not reflect the peak value of the A(t) envelope or the average value, but the magnitude value is related to the pulse repetition frequency.

The output constant voltage of the quasi-peak detector $V_{QP}$ is proportional to the maximum $V_{\max}$ of envelope A(t), and the proportionality coefficient (γ is detection coefficient) is related to the repetition frequency F of the pulse.

(9) $$V_{QP}=\gamma (F)V_{\max},$$

Because $V_{\max}=2E_{i}(f_{0})\cdot g_{\max}$, if $g(t)=G(f_{0})\cdot g_{N}(t)$, $g_{N}(t)$ can be described as,

(10) $$g_{N}(t)=\int_{f1}^{f2}\frac{G(f)}{G(f_{0})}\cdot \cos[\omega t+\varphi (f)]df,$$

In this equation, $G(f_{0})$ is the amplification factor on the centre frequency. 

Because $V_{QP}=\gamma (F)\cdot K(f_{0})\cdot g_{N}{}_{\max}\cdot 2E_{i}(f_{0})$, for sinusoidal waves, if the output of the detector is equal to that of the measured pulse, the following relation applies,

(11) $$V_{QP}=K(f_{0})\cdot \gamma_{\sin}\cdot V,$$

In this equation, V is the true root-mean-square value of the sinusoidal signal which has equal amplitude with the amplitude of the geophone output, where $\gamma_{\sin}$ is the detection coefficient of the sinusoidal signal detector. Thus, we can obtain the value of $V_{QP}=\frac{\gamma (F)}{\gamma_{\sin}}\cdot g_{N\max}\cdot E_{i}(f_{0})$. If $K(f)=\frac{\gamma (F)}{\gamma_{\sin}}\cdot g_{N}{}_{\max}$, it can be observed that this coefficient is only related to the repetition frequency and is independent of the signal voltage, and the output of the signal measurement system is $V_{QP}=K(f)\cdot E_{i}(f_{0})$.

## 4. Data Processing, Analysis, and Results

**Figure 5:**
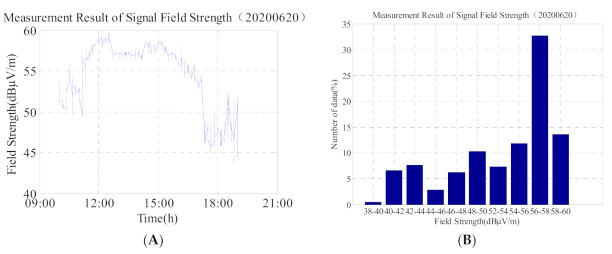
(**A**) Field strength change of low-frequency time-code signal on 20 June 2020; and (**B**) corresponding histogram. Total number of data is 1163.

**Figure 6:**
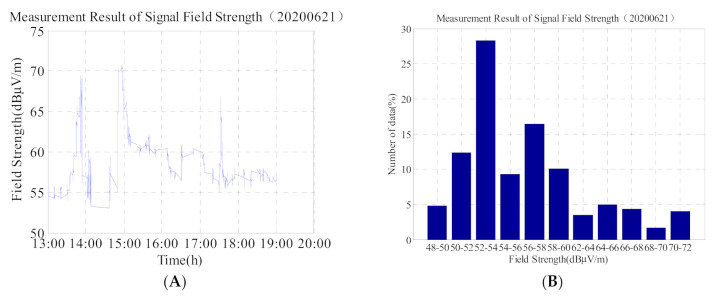
(**A**) Field strength change of low-frequency time-code signal on 21 June 2020 (the day of the annular eclipse) and (**B**) corresponding histogram. Total number of data is 2413.

**Figure 7:**
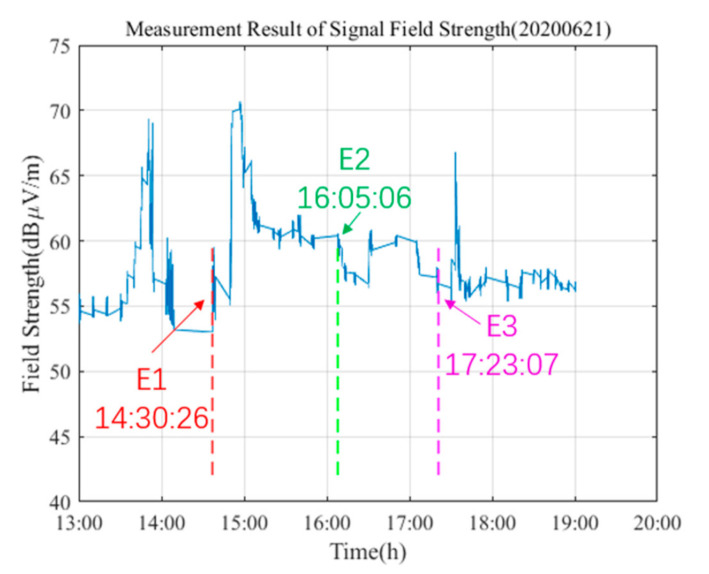
During the process of the annular eclipse, the trend of the field strength variation of the low-frequency time-code signals was observed. The symbols E1, E2, and E3, respectively correspond to the times of first contact, mid totality, and last contact.

**Figure 8:**
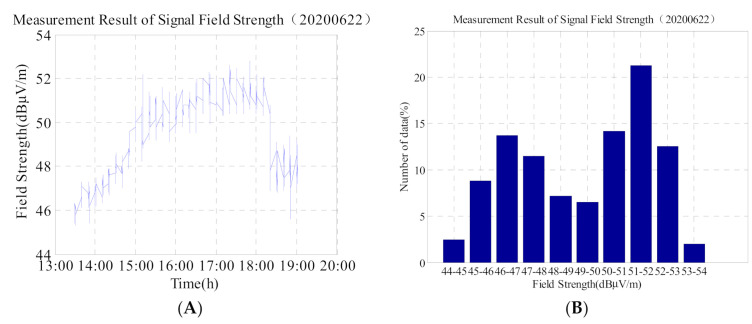
(**A**) Field strength change of the low-frequency time-code signal on 22 June 2020; and (**B**) corresponding histogram. Total number of data is 1104.

**Figure 9:**
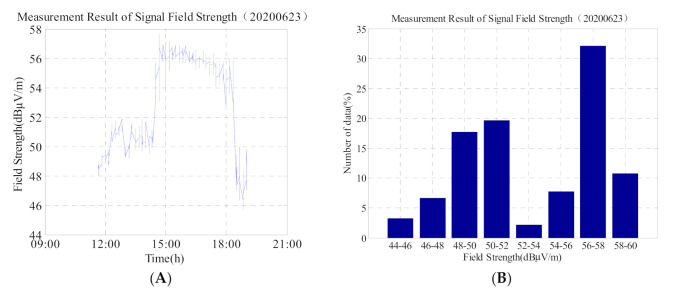
(**A**) Field strength change of the low-frequency time-code signal on 23 June 2020; and (**B**) corresponding histogram. Total number of data is 780.

**Figure 10:**
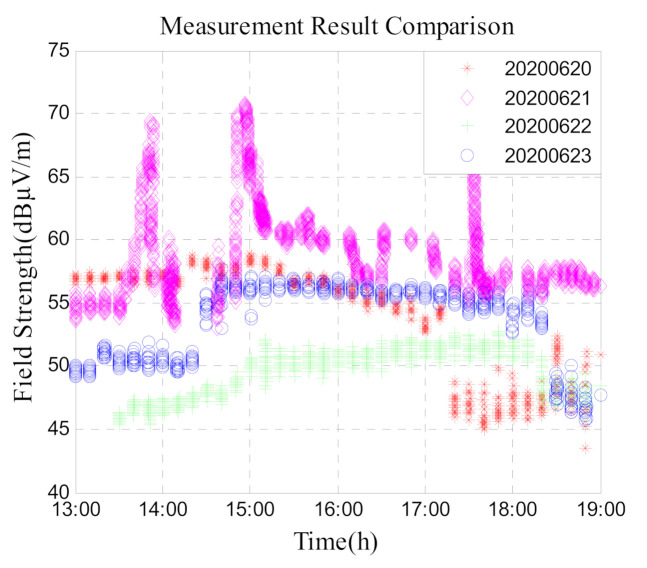
Field strength comparison of low-frequency time-code signal on the day of the annular eclipse and on the days before and after it.

Figure 5, Figure 6, Figure 7, Figure 8, Figure 9 and Figure 10 show the field strength measurement data of the low-frequency time code signal collected between 20 June and 23 June 2020, for 4 days. The test time period was from 03:00 to 11:00 (UTC). To avoid the occurrence of additional interference signals during the test, and ensure the accuracy of the measured data, the testers collected the changes in the field strength of the low-frequency signal before and after the annular eclipse by manually recording the data.

As it can be observed from Figure 5A,B, and Table 2, on 20 June 2020, and the maximum field strength was 59.7 dBμV/m, the minimum field strength was 43.5 dBμV/m, and the difference between the maximum and minimum was 16.2 dBμV/m. Based on the analysis of the above data, the field strength of the low-frequency time-code signal was stable from 04:00–09:00 (UTC). In general, the ionosphere tended to be stable during the time period from 04:00 to 08:30 (UTC), and the field strength of the low-frequency time-code signal varied in the range of 2–3 dBμV/m. The analysis of the measured data on 20 June 2020, shows that the maximum variation of the field strength during the time period from 04:00–06:30 (UTC) on that day was 3.1 dBμV/m, and the standard deviation was 1.1 dBμV/m. Owing to the sunset effect, the signal field strength suddenly dropped at 09:20 (UTC) and slowly increased after 10:50 (UTC).

As it can be observed from Figure 6A,B, and Table 3, there were three sudden changes in the signal on the day of the annular eclipse in the form of a rapid increase in the field strength within a short period of time followed by a slow decline. The time periods of the three sudden changes were 05:45–05:50 (UTC), 06:52–06:57 (UTC), and 09:32–09:35 (UTC), respectively, and the corresponding times of first contact, mid totality, and last contact at the test site were 06:30:26 (UTC), 08:05:06 (UTC), and 09:23:07 (UTC), respectively.

According to the analysis in Figure 7, the first rise of the signal has not yet reached the initial time. Thus, it can be inferred that the ionosphere has changed before the initial of the test point, thus causing the phase of the low-frequency time-code signal to change. Since the low-frequency time-code signal is a superposition of sky waves and ground waves, when the phase difference between the two is zero, the field strength will increase rapidly. Secondly, at the beginning of the eclipse, the moon blocked part of the sun, the electron concentration of the ionosphere and the ionization degree decreased, and the reflection height of the ionosphere increased. This resulted in an abrupt increase in the signal within a short time, and resulted in the second mutation of the signal. As the degree of obscuration increased, when the moon completely blocked the sun, the reflection height of the ionosphere was at its maximum, and the signal field strength also reached its maximum. Subsequently, the signal field strength value gradually decreased, showed a slow oscillation phenomenon, and remained stable. Therefore, it can be observed from Figure 7 that the field strength value after E2 decreased followed by slow oscillatory changes. Finally, when the circular eclipse occurs owing to the fact that the moon no longer blocks the ultraviolet ray’s emission from sun, the ionization level at the ionosphere increases again, and the number of electrons and ions in the ionosphere is gradually increasing. The field strength of the low-frequency time-code signal increases rapidly for the third time and returns to a stable state at a fast rate until the end of the annular eclipse. A review of the entire process of the annular eclipse shows that the dynamic variation range of the low-frequency time-code signal is large, the field strength is very unstable, and the difference between the maximum and minimum values is 17.7 dBμV/m, thus indicating that the ionosphere changes dramatically owing to the blockade of the sun. This has a very obvious influence on the low-frequency time-code signal.

Based on Figure 8A,B, and Table 4, it can be observed that after an annular eclipse occurred on 22 June 2020, a maximum field strength value of 52.8 dBμV/m, a minimum value of 45.3 dBμV/m, and a maximum and minimum difference of 7.5 dBμV/m are documented. Compared with the value on 20 June 2020, the average field strength decreased by approximately 5 dBμV/m. This illustrates (a) the impact of an annular eclipse on the ionosphere, and (b) the fact that the ionosphere is still in a state of flux.

As shown in Figure 9A,B, and Table 5, the field strength of the low-frequency time code signal gradually regressed and stabilised on 23 June with a maximum value of 57.7 dBμV/m and a minimum value of 45.7 dBμV/m. Compared with the data on 20 June, the mean value difference is only 1.5 dBμV/m. Based on the analysis of the above data, it can be concluded that on the third day, after the annular eclipse, the electron concentration and ionization degree in the ionosphere gradually returned to a normal and stable state because the sun was no longer blocked by the moon. At this time, the field strength of the low-frequency time-code signal is also similar to the reference daily change before the solar eclipse.

In addition, we compare the second graph of 5, 6, 8, and 9 and find that: (1) the data on 20 June in Figure 5 is more than 30% in the 56 dBμV/m–58 dBμV/m interval, and the time is concentrated at 03:30–07:30. The ionosphere is relatively stable, so the data does not change much; (2) the data in Figure 6 is widely distributed, and the change range of data is 48 dBμV/m–72 dBμV/m. From the Figure 6, the value which is greater than 60 dBμV/m accounted for about 20%. It shows that on the day of the solar eclipse, although the signal field strength suddenly increased, but the duration was short, and then shows slowly decreased trend; (3) The data value in Figure 8 is relatively lower, but the data distribution range is not wide (44 dBμV/m–53 dBμV/m); (4) The change trend in Figure 8 is similar to that in Figure 5. It can be seen that on the second day after the solar eclipse, the ionosphere gradually returns to the reference day state, and the influence of the solar eclipse is gradually disappearing.

Figure 10 summarizes the four-day data from 20 June to 23 June and compares it. It can be seen from the figure that on 21 June, the day of the solar eclipse, the signal field strength mean value is the largest, and the data trend is more complicated. On the day 22 June, the signal field strength mean value was minimum in a few days, but on 23 June, the data gradually began to rise, the value and change trend of data were similar as the day before the annular solar eclipse (20 June).

## 5. Discussion

The occurrence of an annular eclipse will have a considerable effect on the field strength of the low-frequency time signal. However, the occurrence of solar activities, such as sunspots, solar flares, ionospheric scintillations, and magnetic storms will also have a variety of effects on the radio time signals. Therefore, to explore how the solar eclipse will affect the low-frequency time code signal, and the extent of the impact, it is necessary to analyse the changes in solar activity data, ionospheric data, and geomagnetic data. When the ionospheric and geomagnetic data has no change or the change is small, it can be considered that the change in the field strength of the low-frequency time code signal will only be caused by the occurrence of the solar eclipse, which makes the point of this article more convincing.

### 5.1. Analysis of Solar Activity Data, TEC Data, and Geomagnetic Data

#### 5.1.1. Analysis of Solar Activity Data

Table 6 lists the solar activity data of an annular eclipse during the week of 18–25 June 2020. Based on the analysis of the data, it is observed that the radio traffic is relatively stable, no flare phenomena occur, and the proton and electron fluxes do not exhibit major changes. Thus, it is believed that during the test period, the sun’s activity was low, and there was no violent activity.

#### 5.1.2. Analysis of TEC Data

The total electron content (TEC) of the ionosphere is an important parameter for the study of the ionosphere and for the description of its morphology and structure [27]. TEC is the total number of electrons along a tube of one meter squared cross section [28,29,30]. As time progressed, the blocking effect of the moon was enhanced, and the solar zenith angle decreased [31,32]. The two factors that affected the change of the solar radiation flux to the ionosphere led to D-layer-induced increases of the electron density gradient that was greater than the reference day response value and electron density of the reinforcement. Accordingly, the solar zenith distance decreased, the electron density gradient and the electron density decreased, and then slowly and gradually returned to the reference state at the corresponding time at which the trajectory became circular [33].

**Figure 11:**
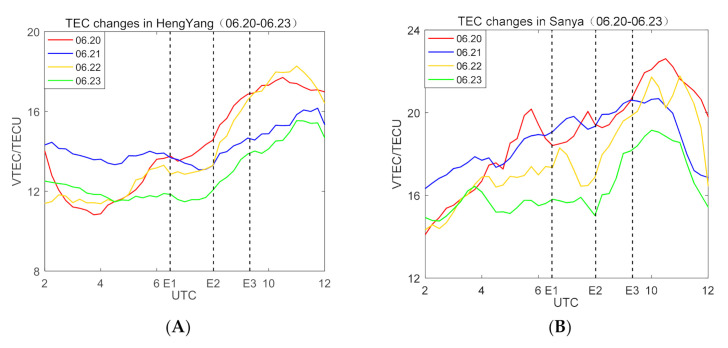
(**A**) The change of Ionospheric Total Electron Content (TEC) from 20 June 2020 to 23 June 2020 in Hengyang, Hunan Province, and (**B**) is TEC change in Sanya, Hainan Province.

Considering that Hengyang is a point on the annularity, it is also the reflection point of the sky wave of the low-frequency time-code signal, so we show the TEC change trend in Figure 11. In 21 June, the TEC content in Hengyang region began to decrease from 02:00 (UTC), and gradually increased from 08:00 (UTC). This change trend is basically the same as the phenomenon described in the previous article. The reason is that the sun is hided, causing the concentration of electrons and ions in the ionosphere to decrease. When the solar eclipse ended, the TEC data gradually began to rise, but it was less than the state of the reference day. However, the TEC content in Sanya region shows different trend. The TEC content in 21 June is slightly higher than the data on the reference day.

#### 5.1.3. Analysis of Geomagnetic Data

In addition to changes in the ionosphere, changes in the geomagnetic field also affect the propagation of radio signals. The geomagnetic field is 600–1000 km above the earth’s surface and is constantly impacted by the solar wind [34]. These charged particles generate a magnetic field and superpose the geomagnetic field. However, during the annular eclipse, owing to the shielding effect of the moon against the sun, the number of the charged particles that shoot into the earth will be significantly reduced, and the geomagnetic field will change accordingly.

In general, the geomagnetic index is a grading index that describes the intensity of geomagnetic disturbances over time, or a physical quantity that describes the intensity of certain types of disturbances (see the earth’s changing magnetic field) divided by the universal time. Geomagnetic indices can be divided into two categories according to their physical meaning [35]. The first index describes the overall level of geomagnetic activity, regardless of the specific types of disturbances, and includes the indices C and Ci, K and Kp, and Ak and Ap. The second type of indices is designed to describe a specific type of magnetic disturbance, including the Dst, AU, AL, and AE indices. We queried the geomagnetic data during the annular eclipse which occurred on 21 June 2020, and we analyzed some of these data to identify the relationship between the geomagnetic data and the ionosphere. Table 7, shows the geomagnetic data statistics from 18–25 June 2020.

1. Dst Data

**Figure 12:**
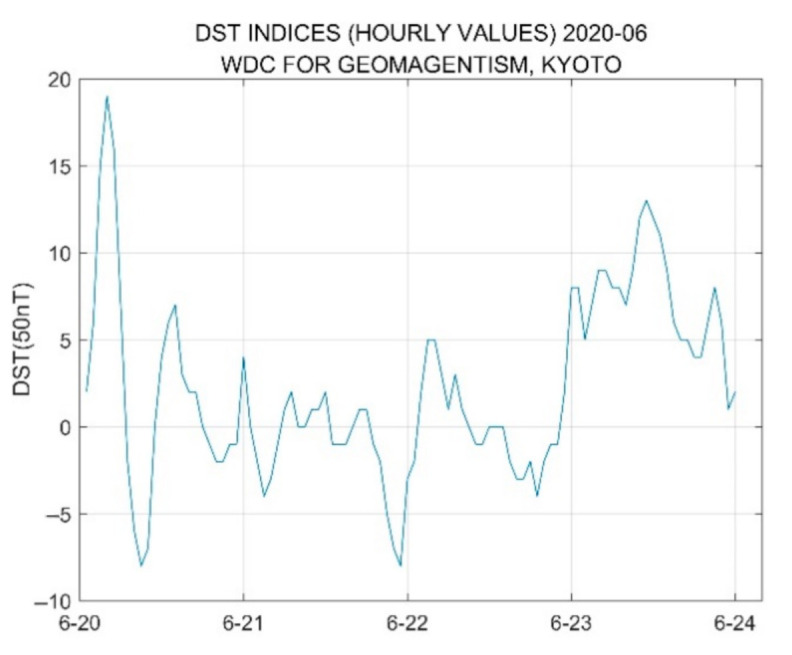
Observation data of Dst from 20 June to 24 June 2020.

The geomagnetic index used at low- and medium-latitude stations is called the Dst index. This index is measured hourly primarily to measure changes in the intensity of the horizontal component of the earth’s magnetism. Figure 12 plots the Dst data collected from 20 June to 24 June. As shown, compared with other days, the Dst index curve on the June 21 is relatively flat, and its extreme values range from −4 to 1 (from 2:00 to 10:00, UTC). Based on this annular eclipse, the level of geomagnetic intensity is less volatile, test points over the region of the ionosphere affected by the solar wind are relatively stable, and no geomagnetic storm occurs.

2. Kp Data

**Figure 13:**
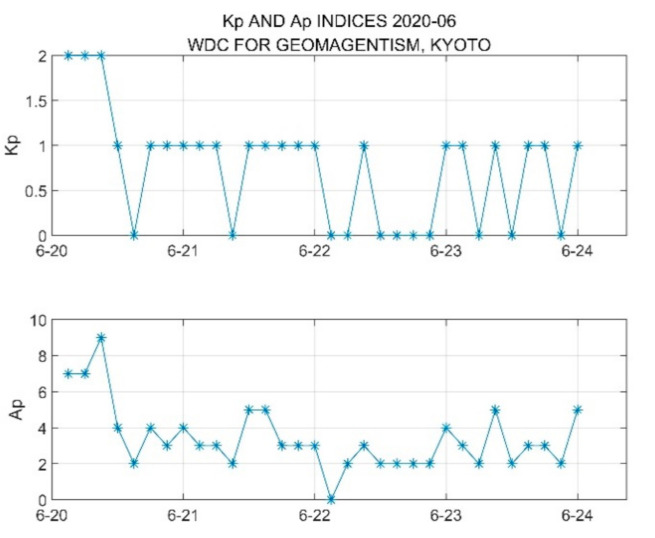
Observation data of Kp from 20 June to 24 June 2020.

The Kp index is used by a single geomagnetic platform to describe the intensity of geomagnetic disturbances every 3 h every day. It is called the 3 h index or the geomagnetic situation index. Based on the analysis of the data plotted in Figure 13, compared to the data of other days, the Kp index was very stable and basically maintained at the value of one throughout the day on 21 June 2020, and we can consider that there was almost no geomagnetic disturbance in the area where the test point was located.

3. Geomagnetic Elements of the Sanya

A geomagnetic element is a physical quantity describing the size and direction of the geomagnetic field at a certain location. In this case, H is the projection of the total strength of the geomagnetic field on the horizontal plane, commonly referred to as the horizontal strength or horizontal component, and D is the angle at which H deviates from the Ox axis, namely the geographic north, which is called the magnetic deviation angle. The eastward deviation of H was positive. Z is the projection of the total intensity of the geomagnetic field on the Oz axis, also known as the vertical intensity or vertical component.

**Figure 14:**
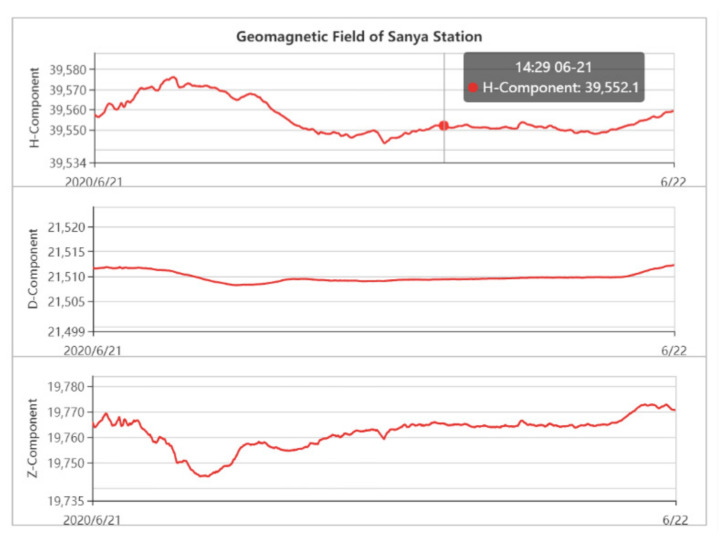
Geomagnetic field data of Sanya collected on 21 June 2020 (source: http://www.sepc.ac.cn/cosmicRays1.php (accessed on 6 February 2021)).

According to the analysis of the data in Figure 14, the H value curve is relatively flat, and minor changes occur during the period of 06:30–09:23 (UTC). Therefore, it can be concluded that the geomagnetic field in the region where the test point is located was not affected considerably by the annular eclipse, the geomagnetic index fluctuated slightly, and the geomagnetic field changed more steadily.

### 5.2. Low-Frequency Time-Code Signal Field Strength Variation Analysis

After the analysis of the solar activity, ionospheric and magnetic field data, we can preliminarily determine the reasons for the variation in the field strength of the low-frequency time-code signal during the annular eclipse:

(1) As the extent of moon shielding gradually increased and then gradually disappeared on the day of the annular eclipse, the total electron content of the ionosphere yielded a decreasing trend first followed by an increasing trend [36,37]. This was the main reason for the variation in the field strength of the low-frequency time-code signal
(2) On 21 June the solar activity and geomagnetic data analysis indicated that, on that day, the sun and the earth’s magnetic field activities were relatively stable, and no solar flares or geomagnetic storm phenomena occurred. Therefore, the occurrence of dramatic changes in the low-frequency field strength signal attributed to the solar activity or the magnetic field caused by the change can be dismissed
(3) After the exclusion of the influences of solar activity and geomagnetic field changes on the intensity low-frequency time-code signal, the reason for the change in signal field strength can be considered as the impact of the annular eclipse on the ionosphere. When an annular eclipse occurs, because the sun is hidden, there is a low degree of ionisation (ions and electrons) in the ionosphere, and the ionospheric healing gradually accelerates. Long-distance transmissions have a dependence on the ionosphere for reflection. Therefore, in the low-frequency time-code signal propagation path, the height of lower ionosphere increased, the signal attenuation was small and the signal reaching the ground shows an enhanced state. After the eclipse, the density of charged particles shot by the sun to the earth recovered again because of the influence of the moon’s shade, the reflection height of the low ionosphere decreased gradually, and the signal field strength returned at a slow rate to its reference day value.

## 6. Conclusions

This study set up a signal parameter measurement system in Sanya, Hainan Province, and used the low-frequency time-code signal as an example for the annular eclipse that occurred on 21 June 2020. Accordingly, a detailed test was conducted on the field strength of the low-frequency time-code signal along the path of Shangqiu–Hengyang–Sanya. Based on a detailed analysis of the measured data, the test group can draw the following conclusions:

(1) In the early solar eclipse, the low-frequency time-code signals changed dramatically, and the signal field strength yielded ‘three rises and three falls’. The reason for this was attributed to the fact that at the beginning of the deficit, the moon began to block the sun and led to a decrease in the degree of ionization of the ionosphere, a decrease in electron concentration, and an increase in the reflection height of the ionosphere, that resulted in an abrupt increase in the signal within a short period of time and to a sudden change in the signal. Additionally, the electron density, reflection coefficient, and field strength decreased followed by a slow oscillation change, and finally achieved a stable state. When the trajectory was circular (because the moon no longer blocked the sun), the ionization level of the ionosphere increased again, and the ionosphere became gradually active. At low frequencies, the field strength of the code signal increased rapidly for the third time, and then fell back to a stable state until the end of the annular eclipse.
(2) In addition, combined with the analysis of solar activity, ionospheric, and geomagnetic data, it was found that the solar activity became calm, and no magnetic storm phenomena occurred. Therefore, it could be inferred that the dramatic change in the field strength was caused by the change in the ionosphere which was caused by the different stages of the annular eclipse.
(3) In the experiment, the low-frequency time-code signal exhibited an extremely unstable state during the solar eclipse, and the signal field strength increased abruptly three times and dropped three times. The average rates of change were 2.4, 3.1, and 3.3 dBμV/m·min based on the above data. The signal field strength and coverage were affected by the annular eclipse. Accordingly, the problem that needs to be considered next relates to the efforts that ought to be expended to ensure that the signal of the low-frequency time-code system is not affected when the ionosphere changes dramatically.

## Figures and Tables

**Table 1:** Information related to the signal transmission (Shangqiu, Henan Province) and reception points (Sanya, Hainan Province) and sky wave reflection points (Hengyang, Hunan Province) during the annular eclipse on 21 June 2020 (source: http://www.mnw.cn/news/china/2292341.html (accessed on 6 February 2021)).

Location	Coordinates	First Contact (UTC)	Mid Totality (UTC)	Last Contact (UTC)	Degree of Obscuration
Shangqiu, Henan Province	E 109°32.574′ N 34°56.915′	06:32:17	07:56:25	09:09:29	0.744
Hengyang, Hunan Province	E 112°23.851′ N 26°25.060′	06:28:44	08:01:31	09:19:33	0.991
Sanya, Hainan Province	E 108°59.393′ N 18°21.909′	06:30:26	08:05:06	09:23:07	0.725

**Table 2:** Statistical values of the low-frequency time-code signal on 20 June 2020.

Test Period (UTC)	Mean Value (dBμV/m)	Maximum (Max) Value (dBμV/m)	Minimum (Min) Value (dBμV/m)	Standard Deviation (STD) (dBμV/m)
03:00:00–11:00:34	54.2785	59.7	43.5	3.9913

**Table 3:** Statistical value of the low-frequency time-code signal field strength on 21 June 2020 (the day of the annular eclipse).

Test Period (UTC)	Mean Value (dBμV/m)	Max Value (dBμV/m)	Min Value (dBμV/m)	STD (dBμV/m)
04:00:00–11:00:48	59.5872	70.7	53	11.3475

**Table 4:** Statistics of the low-frequency time-code signal on 22 June 2020.

Test Period (UTC)	Mean Value (dBμV/m)	Max Value (dBμV/m)	Min Value (dBμV/m)	STD (dBμV/m)
05:00:00–11:01:02	49.3145	52.8	45.3	1.8550

**Table 5:** Statistics of the low-frequency time-code signal on 23 June 2020.

Test Period (UTC)	Mean Value (dBμV/m)	Max Value (dBμV/m)	Min Value (dBμV/m)	STD (dBμV/m)
03:40:00–11:00:28	52.7680	57.7	45.7	3.1480

**Table 6:** Solar activity data during the period of 18–25 June 2020. (source: http://www.geomag.bgs.ac.uk/data_service/space_weather/home.html (accessed on 6 February 2021)).

Date	Radio Traffic 10.7 cm	Sunspot Numbers	X-ray Background	Flare							GOES1310 MeV Protons (cm^2^·day·sr)	GOES132 MeV Electrons (cm^2^·Day·sr)	**Ap Index**
				X-ray Flares				Optical Flares					
				C	M	X	S	1	2	3			
18 June 2020	68	0	<A1.0	0	0	0	0	0	0	0	4.70 × 10^4^	1.20 × 10^6^	4
19 June 2020	69	0	<A1.0	0	0	0	0	0	0	0	4.70 × 10^4^	1.20 × 10^6^	5
20 June 2020	68	0	<A1.0	0	0	0	0	0	0	0	4.70 × 10^4^	1.30 × 10^6^	6
21 June 2020	68	0	<A1.0	0	0	0	0	0	0	0	4.70 × 10^4^	1.40 × 10^6^	4
22 June 2020	68	0	<A1.0	0	0	0	0	0	0	0	4.70 × 10^4^	1.30 × 10^6^	4
23 June 2020	67	0	<A1.0	0	0	0	0	0	0	0	4.70 × 10^4^	1.20 × 10^6^	4
24 June 2020	67	0	<A1.0	0	0	0	0	0	0	0	4.70 × 10^4^	1.20 × 10^6^	5

**Table 7:** Geomagnetic data statistics during the period of 18–25 June 2020 (source: http://www.geomag.bgs.ac.uk/data_service/space_weather/solar.html (accessed on 6 February 2021)).

Date	Kp: Planetary 3 h Range Index								Sum Kp	Ap: Planetary Equivalent Amplitude								**Ap**	**Cp**	**C9**
18 June 2020	3	17	10	10	10	7	10	7	73	2	6	4	4	4	3	4	3	4	0.1	0
19 June 2020	7	10	3	10	7	7	13	17	73	3	4	2	4	3	3	5	6	4	0.1	0
20 June 2020	20	20	23	10	3	10	7	10	103	7	7	9	4	2	4	3	4	5	0.2	1
21 June 2020	7	7	3	13	13	7	7	7	63	3	3	2	5	5	3	3	3	3	0.1	0
22 June 2020	0	3	7	3	3	3	3	10	33	0	2	3	2	2	2	2	4	2	0.0	0
23 June 2020	7	3	13	3	7	7	3	13	57	3	2	5	2	3	3	2	5	3	0.1	0
24 June 2020	10	10	7	10	7	7	3	7	60	4	4	3	4	3	3	2	3	3	0.1	0
25 June 2020	7	7	3	3	3	7	3	10	43	3	3	2	2	2	3	2	4	3	0.0	0

## Data Availability

The solar activity data and geomagnetic data (Table 6 and Table 7, and Figure 14) in this article come from 3rd Party Data.
